# Draft genome sequence of cauliflower (*Brassica oleracea* L. var. *botrytis*) provides new insights into the C genome in *Brassica* species

**DOI:** 10.1038/s41438-019-0164-0

**Published:** 2019-07-01

**Authors:** Deling Sun, Chunguo Wang, Xiaoli Zhang, Wenlin Zhang, Hanmin Jiang, Xingwei Yao, Lili Liu, Zhenghua Wen, Guobao Niu, Xiaozheng Shan

**Affiliations:** 1 Tianjin Academy of Agricultural Sciences, 300192 Tianjin, China; 2 College of Life Sciences, Nankai University, 300071 Tianjin, China; 3 Tianjin Kernel Vegetable Research Institute, 300384 Tianjin, China; 4 Novogene Bioinformatics Institute, 100015 Beijing, China

**Keywords:** Plant evolution, Genome assembly algorithms

## Abstract

Cauliflower is an important variety of *Brassica oleracea* and is planted worldwide. Here, the high-quality genome sequence of cauliflower was reported. The assembled cauliflower genome was 584.60 Mb in size, with a contig N50 of 2.11 Mb, and contained 47,772 genes; 56.65% of the genome was composed of repetitive sequences. Among these sequences, long terminal repeats (LTRs) were the most abundant (32.71% of the genome), followed by transposable elements (TEs) (12.62%). Comparative genomic analysis confirmed that after an ancient paleohexaploidy (γ) event, cauliflower underwent two whole-genome duplication (WGD) events shared with *Arabidopsis* and an additional whole-genome triplication (WGT) event shared with other *Brassica* species. The present cultivated cauliflower diverged from the ancestral *B. oleracea* species ~3.0 million years ago (Mya). The speciation of cauliflower (~2.0 Mya) was later than that of *B. oleracea* L. var. *capitata* (approximately 2.6 Mya) and other *Brassica* species (over 2.0 Mya). Chromosome no. 03 of cauliflower shared the most syntenic blocks with the A, B, and C genomes of *Brassica* species and its eight other chromosomes, implying that chromosome no. 03 might be the most ancient one in the cauliflower genome, which was consistent with the chromosome being inherited from the common ancestor of *Brassica* species. In addition, 2,718 specific genes, 228 expanded genes, 2 contracted genes, and 1,065 positively selected genes in cauliflower were identified and functionally annotated. These findings provide new insights into the genomic diversity of *Brassica* species and serve as a valuable reference for molecular breeding of cauliflower.

## Introduction

The genus *Brassica* contains three basic genomes (A, B and C) that form three diploid species, namely, *Brassica rapa* (AA genome), *Brassica nigra* (BB genome), and *Brassica oleracea* (CC genome), which further hybridize to give rise to three allopolyploid species, namely, *Brassica napus* (AACC genome), *Brassica juncea* (AABB genome), and *Brassica carinata* (BBCC genome), as described by the triangle of U model^1^. These *Brassica* species encompass many important vegetable and oilseed crops, such as Chinese cabbage, turnip, cabbage, broccoli, cauliflower, and oilseed rape. Among them, cauliflower (*B. oleracea* L. var. *botrytis*, CC genome) is an important variety of *B. oleracea* that differs from most *Brassica* species in its formation of a specialized organ called the curd during floral development^2^. Curds are composed of many indeterminate inflorescences and shortened inflorescence branches^3,4^ and are the primary edible organs of cauliflower, with abundant nutrient materials, such as sulforaphane^5^. Sulforaphane is regarded as one of the most powerful natural bioactive substances in preventing and/or defending against cancers^6–10^. In the past decade, the genomes of several *Brassica* species, including the *B. rapa* cultivar *Chiifu*^11^; *B. oleracea* cultivars *02-12*^12^, *TO1000DH*^13^, and *HDEM*^14^; *B. nigra* cultivar *YZ12151*^15^; *B. napus* cultivars Darmor*-bzh*^16^, *ZS11*^17^, and *Tapidor*^18^; and *B. juncea* cultivar *T84-66*^15^, have been explored. These studies provided insights into the evolution and phenotypic diversification of *Brassica* species. However, our knowledge of genome variation and agriculturally important trait formation in cauliflower, one of the most important vegetable crops, is still lacking. Here, the genome of cauliflower was sequenced by PacBio and Illumina sequencing technologies to further understand the evolution of *Brassica* species, especially the genetic variation in the C genome of *B. oleracea* species, and reveal the formation of extreme morphological characteristics, namely, the enlarged inflorescences (curds).

## Materials and methods

### Plant materials

An advanced-generation inbred line of *B. oleracea* L. var. *botrytis* (C-8) was selected for whole-genome sequencing, which is widely used as a parental line for breeding due to its excellent agronomic traits. Ten-day-old seedlings of C-8 were harvested and stored at −80 °C prior to DNA extraction.

### Genome sequencing

Genomic DNA was extracted from the 10-day-old seedlings of C-8 by using a DNA Secure Plant Kit (TIANGEN, China) and broken into random fragments. DNA sequencing libraries were constructed in accordance with the standard Illumina library preparation protocols. Paired-end libraries with insert sizes of 350 bp were constructed according to the manufacturer’s instructions (Illumina, CA, USA). All of the constructed libraries were sequenced on an Illumina HiSeq X Ten. At least 10 μg of sheared DNA is required to construct PacBio libraries. SMRTbell template preparation involved DNA concentration, damage repair, end repair, hairpin adapter ligation, and template purification. SMRTbell libraries with an insert size of 20 kb were constructed and then sequenced on a PacBio Sequel platform (Pacific Biosciences, CA, USA) by using P6 polymerase/C4 chemistry in accordance with the manufacturer’s procedure (Pacific Biosciences, CA, USA).

### Estimation of genome size

Genome size was estimated by K-mer distribution analysis. Approximately 45 Gb of high-quality paired-end reads (350 bp) was generated and used to determine the abundance of 17-nt K-mers. The distribution of 17-nt K-mers depends on the characteristics of the genome and follows a Poisson distribution.

### Genome assembly and quality evaluation

The de novo assembly of PacBio single-molecule long reads from Single Molecule Real Time (SMRT) sequencing was performed by using FALCON^19^ (https://github.com/PacificBiosciences/FALCON/). The 60 subreads with the longest coverage were first selected as seed reads for error correction to obtain enough corrected reads. Then, the error-corrected reads were aligned to one another and assembled into genomic contigs by using FALCON with the following parameters: length_cutoff_ pr = 5,000, max_diff = 120, and max_cov = 130. The draft assembly was polished using the Quiver algorithm. Subsequently, Pilon^20^ was used to perform error correction of p-contigs with the short paired-end reads generated from an Illumina HiSeq platform. The draft assembly was evaluated by mapping the high-quality reads from short-insert-size libraries to the contigs by using BWA-MEM^21^. The distribution of the sequencing depth at each position was calculated with SAMtools^22^ to assess the completeness of the assembled genome. The GC content distribution of the assembled genome was examined to analyze the nucleotide distribution, assess the randomness of sequencing, and check for possible sample contamination. The Core Eukaryotic Genes Mapping Approach (CEGMA)^23^ pipeline was used to assess the completeness of the genome assembly, which was further assessed by Benchmarking Universal Single-Copy Orthologs (BUSCO)^24^ analysis.

### Transcriptome sequencing

Four tissues of C-8 (leaf, curd, flower and root) were collected for RNA-seq analysis, and four sequencing libraries were constructed from these tissues using an Illumina standard mRNA-seq prep kit.

### Genome annotation

TEs and other repetitive sequences in the C-8 genome were searched by combining de novo- and homology-based approaches. For the de novo-based approach, RepeatModeler^25^, LTR_FINDER^26^, and RepeatScout^27^ were used to build a de novo repeat library. For the homology-based approach, the Repbase TE library and the TE protein database were searched against the cauliflower genome by utilizing RepeatMasker (version 3.3.0)^28^ and RepeatProteinMask, respectively. Tandem repeats were detected in the genome by using Tandem Repeats Finder software^29^.

Homology-based prediction and de novo-based prediction combined with transcriptome-based prediction were conducted to predict the protein-coding genes in the cauliflower genome. Homologous proteins from six plant genomes (*Arabidopsis thaliana*, *Arabidopsis lyrata*, *B. oleracea*, *B. rapa*, *Capsella rubella*, and *Raphanus sativus*) were downloaded from the Ensembl and NCBI databases. The protein sequences from these genomes were aligned to the cauliflower genome assembly by using TBLASTN^30^ with an E-value cutoff of 1e-05. BLAST hits were conjoined by Solar software^31^. GeneWise^32^ was used to predict the exact gene structure of the corresponding genomic regions for each BLAST hit (Homology-set). For transcriptome-based prediction, RNA-seq data were mapped to the assembly by using TopHat (version 2.0.8)^33^, and Cufflinks (version 2.1.1)^34^ (http://cufflinks.cbcb.umd.edu/) was then used to assemble the transcripts into gene models (Cufflinks-set). In addition, the RNA-seq data were assembled by Trinity^35^ to create several pseudo-ESTs. These pseudo-ESTs were also mapped to the assembly, and gene models were predicted by PASA^36^. This gene set was denoted PASA-T-set (PASA Trinity set) and used to train *ab initio* gene prediction programs. Five *ab initio* gene prediction programs, namely, Augustus (version 2.5.5)^37^, GENSCAN (version 1.0)^38^, GlimmerHMM (version 3.0.1)^39^, geneid^40^, and SNAP^41^, were used to predict the coding regions in the repeat-masked genome. Gene model evidence from the Homology-set, Cufflinks-set, PASA-T-set and *ab initio* programs were combined by EVidenceModeler (EVM)^42^ into a nonredundant set of gene structures.

Functional annotation of the protein-coding genes was achieved using BLASTP (E-value of 1e-05)^43^ against two integrated protein sequence databases: SwissProt and NR. Protein domains were annotated by searching against the InterPro (V32.0)^44^ and Pfam (V27.0)^45^ databases, using InterProScan (V4.8)^46^ and HMMER (V3.1)^47^, respectively. The Gene Ontology (GO) terms^48^ for each gene were obtained from the corresponding InterPro or Pfam entry. The pathways in which the genes might be involved were assigned by BLAST against the Kyoto Encyclopedia of Genes and Genomes (KEGG) database (release 53)^49^, with an E-value cutoff of 1e-05.

The tRNA genes were identified by tRNAscan-SE software^50^. The rRNA fragments were predicted by aligning the rRNA sequences by using BLASTN at an E-value of 1e-10. The miRNAs and snRNAs were predicted by INFERNAL software^51^ against the Rfam database (release 9.1)^52^.

### Gene family construction

The protein sequences from cauliflower and other sequenced plant genomes with representatives from *A. thaliana*, *A. lyrata*, *Aethionema arabicum*, *B. juncea* (AABB genome), *B. napus Darmor-bzh* (AACC genome), *B. napus ZS11* (AACC genome), *B. napus Tapidor* (AACC genome), *B. nigra* (BB genome), *B. oleracea* L. var. *capitata* (CC genome), *B. oleracea TO1000DH* (CC genome), *B. rapa* (AA genome), *Cardamine hirsuta*, *C. rubella*, *Eutrema salsugineum*, *Sisymbrium irio* and *Schrenkiella parvula* were used for gene family clustering. The gene set of each species was filtered as follows: first, when multiple transcripts were present in one gene, only the longest transcript in the coding region was obtained for further analysis; second, the genes encoding proteins of fewer than 30 amino acids were filtered out. Then, the similarity relation between the protein sequences of all of the species was obtained through BLASTP with an E-value of 1e-05. All of the protein datasets of the representative plant species were clustered into paralogs and orthologs by using the program OrthoMCL (http://orthomcl.org/orthomcl/)^53^ with an inflation parameter of 1.5. The three members of *Brassica* with a C genome, namely, cauliflower, *B. oleracea* L. var. *capitata* and *B. oleracea TO1000DH*, were further clustered and subjected to Venn diagram analysis to explore the species-specific genes in cauliflower. The cauliflower-specific genes were then subjected to GO and KEGG functional annotation.

### Phylogenetic tree and divergence time estimation

The single-copy genes in the representative plant genomes were aligned by MUSCLE (http://www.drive5.com/muscle/)^54^, and the alignment results were combined to create a super-alignment matrix. A phylogenetic tree comprising cauliflower and other representative plant species was constructed by using RAxML (http://sco.h-its.org/exelixis/web/software/raxml/index.html) with the maximum likelihood method, and the number of bootstraps was 1,000^55^. *A. arabicum* was designated as the outgroup of the phylogenetic tree. The MCMCTree program of PAML5 (http://abacus.gene.ucl.ac.uk/software/paml.html) was applied to infer the divergence time based on the constructed phylogenetic tree, employing the following parameters: burn-in = 5,000,000, sample number = 1,000,000, and sample frequency = 50^56^. The calibration time of divergence of these plant species was obtained from the TimeTree database (http://www.time.org/)^57^.

### Detection of polyploidization events

The protein sequences from cauliflower, *B. nigra*, *B. napus* Darmor*-bzh*, *B. oleracea TO1000DH*, and *B. rapa* were searched against themselves by using BLASTP (E-value < 1e-05) to identify syntenic blocks. The results were also subjected to MCScanX^58^ to determine the syntenic blocks. At least five genes were required to indicate synteny. Subsequently, the protein sequence alignments were converted into a CDS file, and 4DTv values were calculated on the basis of CDS alignments accompanying the correction of the HKY model.

### Gene family expansion and contraction analysis

The expansion and contraction of the gene families were analyzed by comparing the cluster size differences between the ancestor and each species by using the CAFÉ program^59^. A random birth and death model was used to study the changes in gene families along each lineage of the phylogenetic tree. A probabilistic graphical model (PGM) was introduced to calculate the probability of transitions in gene family size from parent to child nodes in the phylogeny. Using conditional likelihoods as the test statistics, the corresponding *p*-value in each lineage was calculated, and a *p*-value of 0.05 was used to identify the families that were significantly expanded or contracted. The expanded and contracted genes were then subjected to GO and KEGG functional annotation.

### Positively selected gene analysis

The single-copy genes from cauliflower and two other relative species were aligned using MUSCLE^54^ to identify the positively selected genes in cauliflower compared with other *Brassica* species with the C genome. Likelihood ratio tests based on the branch-site models of PAML^56^ were conducted to detect positive selection, with cauliflower as a foreground branch. *p* values were computed using the χ^2^ statistic and corrected for multiple testing by the false discovery rate (FDR) method. The positively selected genes were subjected to GO and KEGG functional annotation.

## Results

### Genome sequencing and assembly

The cauliflower genome estimated by K-mer analysis was 603.04 Mb. A total of 69.06 Gb of high-quality PacBio long reads (114.52X coverage of the genome) and 45.99 Gb of Illumina clean reads (76.26X coverage of the genome) were generated, resulting in approximately 190.78-fold coverage of the cauliflower genome. All of these reads were further assembled into 584.60 Mb, consisting of 1,484 contigs with a contig N50 of 2.11 Mb and a longest contig of 9.81 Mb, which represented 96.94% of the cauliflower genome (Table 1). The high-quality reads from the short-insert paired-end libraries were mapped to the contigs by BWA-MEM to evaluate the quality of the assembled genome. Approximately 99.43% of the reads could be mapped to the assembly, which covered 99.15% of the assembled sequence (Table S1). The CEGMA and BUSCO pipelines were used to further assess the completeness of the genome assembly. The CEGMA results confirmed the homologs for 96.77% of the core eukaryotic genes in the assembly (Table S2). BUSCO analysis revealed that 97.2% of the genes in the cauliflower genome were conserved. These results verified the high quality of the presently generated cauliflower genome assembly (Table S3). The raw genome and transcriptome sequencing data are available from the NCBI under the project ID PRJNA516731.

**Table 1:** Statistics and annotated analysis of the cauliflower genome assembly

	Number	Size	Sequence coverage (X)	Percentage
Estimate of genome size		603.04 Mb		
PacBio reads		69.06 Gb	114.52	
Illumina reads		45.99 Gb	76.26	
Total reads		115.05 Gb	190.78	
Contigs	1,484	584.60 Mb		
Coverage of sequenced genome				96.94 %
N50 of contigs	82	2.11 Mb		
Longest contig		9.81 Mb		
GC content				36.76 %
Total repetitive sequences		331.20 Mb		56.65 %
Total protein-coding genes	47,772	108.40 Mb		18.54 %
Annotated protein-coding genes	46,628			97.60 %
Average length per gene (exon + intron)		2,035 bp		
Average exons per gene	4.78	242 bp		
Average length per intron		260 bp		
Noncoding RNAs	8,106	1.32 Mb		0.23 %

### Genome annotation

The combined de novo-, homology-, and transcriptome-based predictions revealed that 108.40 Mb of the 584.60-Mb assembled genome (18.54% of the genome) was annotated to code 47,772 genes, with 4.78 exons per gene on average. The average transcript length per gene was 2,035 bp, with average exon and intron lengths of 242 and 260 bp per transcript, respectively. Among the 47,772 predicted genes, 46,628 genes (97.60% of the total genes) could be annotated in at least one functional protein database. The de novo-based and homology-based approaches were also used to search for and predict repetitive sequences. A total of 56.65% of the assembled cauliflower genome was composed of repetitive sequences (Table 1). Among these repetitive elements, LTRs were the most abundant, accounting for 32.71% of the genome, followed by TEs (12.62%), tandem repeats (9.34%), LINEs (5.20%), simple repeats (2.89), and SINEs (0.06%) (Table S4). In addition, 0.23% of the assembled genome was annotated as noncoding RNAs, which included 1,828 miRNAs, 1,408 tRNAs, 2,420 rRNAs, and 2,450 snRNAs (Table S5).

### Evolution of the cauliflower genome

**Fig. 1:**
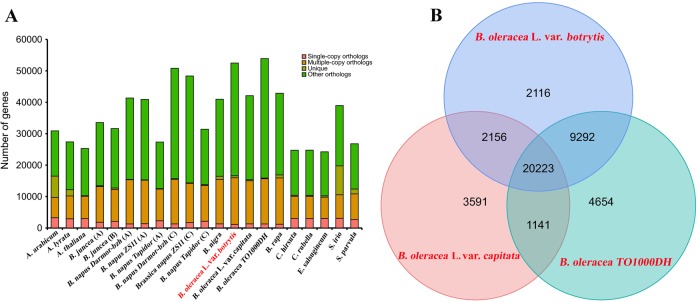
Distribution of genes in cauliflower and other representative plant species. **a** Distribution of single-copy orthologs (pink), multiple-copy orthologs (orange), unique genes (brown), and other unclassified genes (green) in cauliflower and other representative plant species. **b** Venn diagram of gene families in cauliflower and two other *Brassica* species with the C genome

**Fig. 2:**
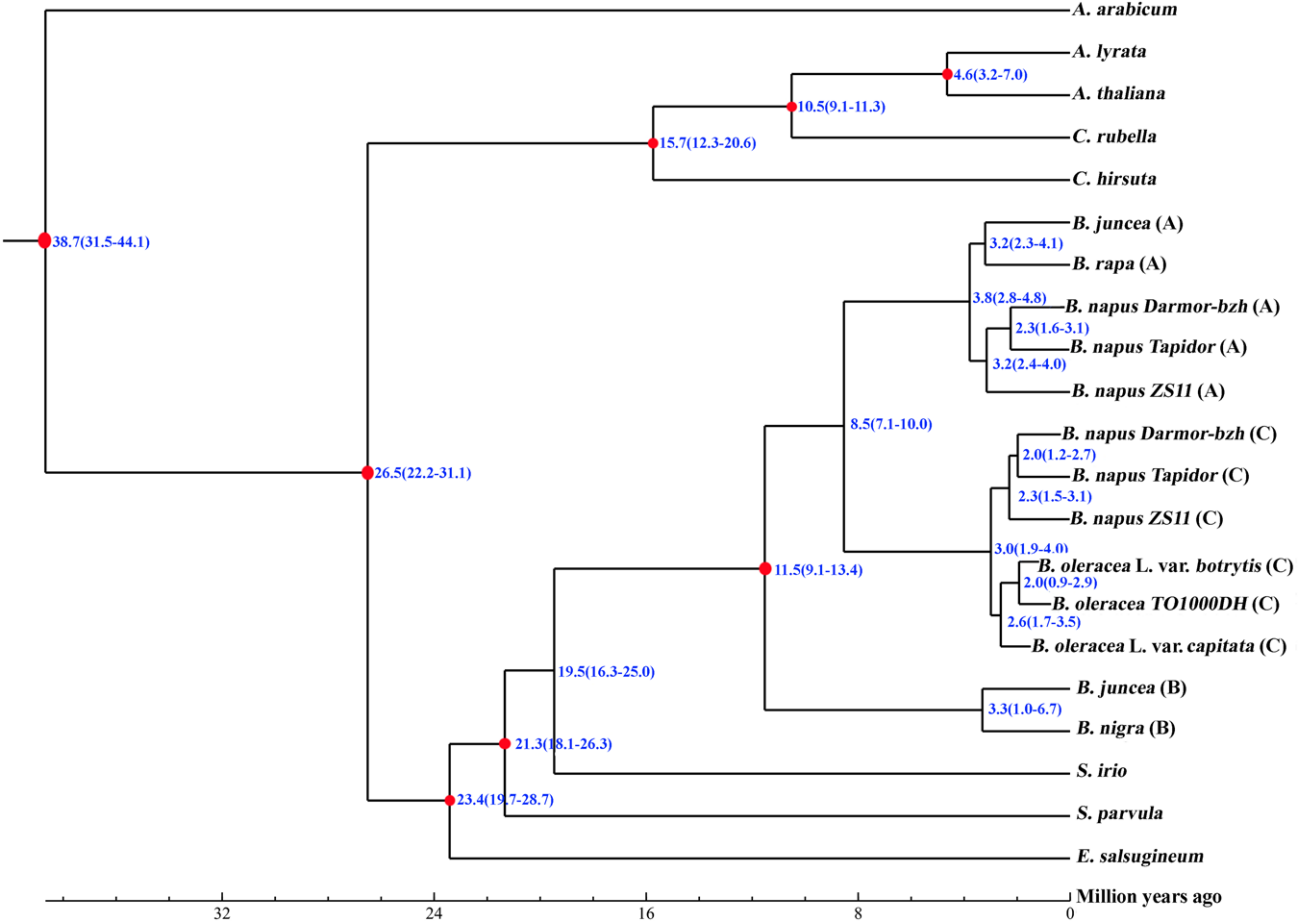
Divergence time of cauliflower and other representative plant species. The nodes represent the divergence time from present (million years ago, Mya). The blue number in the brackets indicates the confidence interval of the divergence time. **a–c** indicate the **A**, **B** and **C** genomes of *Brassica* species, respectively

The genome sequences of the representative plant species were collected and subjected to comparative genomic analysis with cauliflower to reveal the genome evolution and divergence of cauliflower. The clustering results revealed 64,592 gene families in these plant species, and among them, 3,109 gene families were common. Furthermore, 434 of the 3,109 common gene families contained one copy in each plant species (Fig. 1a). These 434 single-copy orthologous genes were used to construct the phylogenetic tree. The results confirmed that the A, B, and C genomes from different *Brassica* species were classified into three independent branches, consistent with the results described in the triangle of U model. *Brassica* species with A and C genomes displayed a shorter genetic distance than the species with the B genome. In the three *Brassica* species with the C genome, cauliflower and *B. oleracea TO1000DH* were clustered together on a branch (Figure S1). Furthermore, the molecular clock of these plant genomes was calculated. The calibration times of divergence were 3.2–7.0 Mya between *A. lyrata* and *A. thaliana*, 9.1–11.3 Mya between *A. thaliana* and *C. rubella*, 12.3–20.6 Mya between *C. rubella* and *C. hirsuta*, and 18.1–26.3 Mya between *S. parvula* and *S. irio* (Fig. 2). The data further confirmed that the *Brassica* ancestor diverged from *S. irio* approximately 16.3–25.0 Mya. In *Brassica* species, the diploid species *B. nigra* with the B genome diverged from the A and C genomes approximately 9.1–13.4 Mya. Then, the other two diploid species, namely, *B. oleracea* (CC genome) and *B. rapa* (AA genome), diverged ~8.5 Mya. Furthermore, the A genome of diploid *B. rapa* diverged into the A subgenome of allotetraploid *B. juncea* 2.3–4.1 Mya and *B. napus* 2.8–4.8 Mya. The three subgenomes of *B. napus* diverged 2.4–4.0 Mya. The C genome of the *B. oleracea* ancestral species diverged into the C subgenome of allotetraploid *B. napus*, uncultivated *B. oleracea TO1000DH* (wild cabbage) and two varieties, specifically, cauliflower and *B. oleracea* L. var. *capitata*, approximately 3.0 Mya. *B. oleracea TO1000DH* diverged from the variety *B. oleracea* L. var. *capitata* approximately 2.6 Mya, whereas the divergence time between cauliflower and *B. oleracea TO1000DH* was ~2.0 Mya (Fig. 2).

**Fig. 3:**
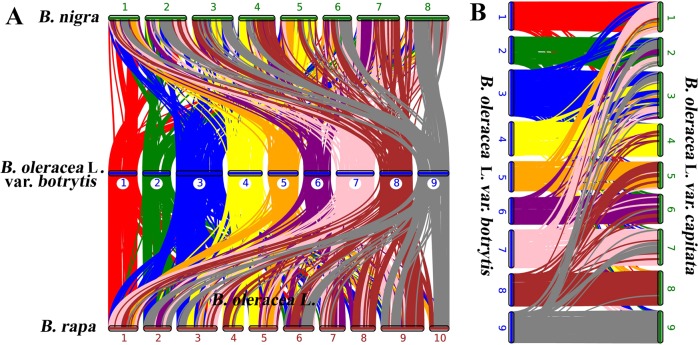
Synteny analysis of genes in cauliflower, *B. nigra*, *B. rapa* and *B. oleracea* L. var. *capitata.* **a** Syntenic blocks of cauliflower with *B. nigra* and *B. rapa*. **b** Syntenic blocks of cauliflower with *B. oleracea* L. var. *capitata*. The numbers indicate the corresponding chromosomes in each species. The detailed syntenic blocks and the associated genes are shown in Tables S6–S13

**Fig. 4:**
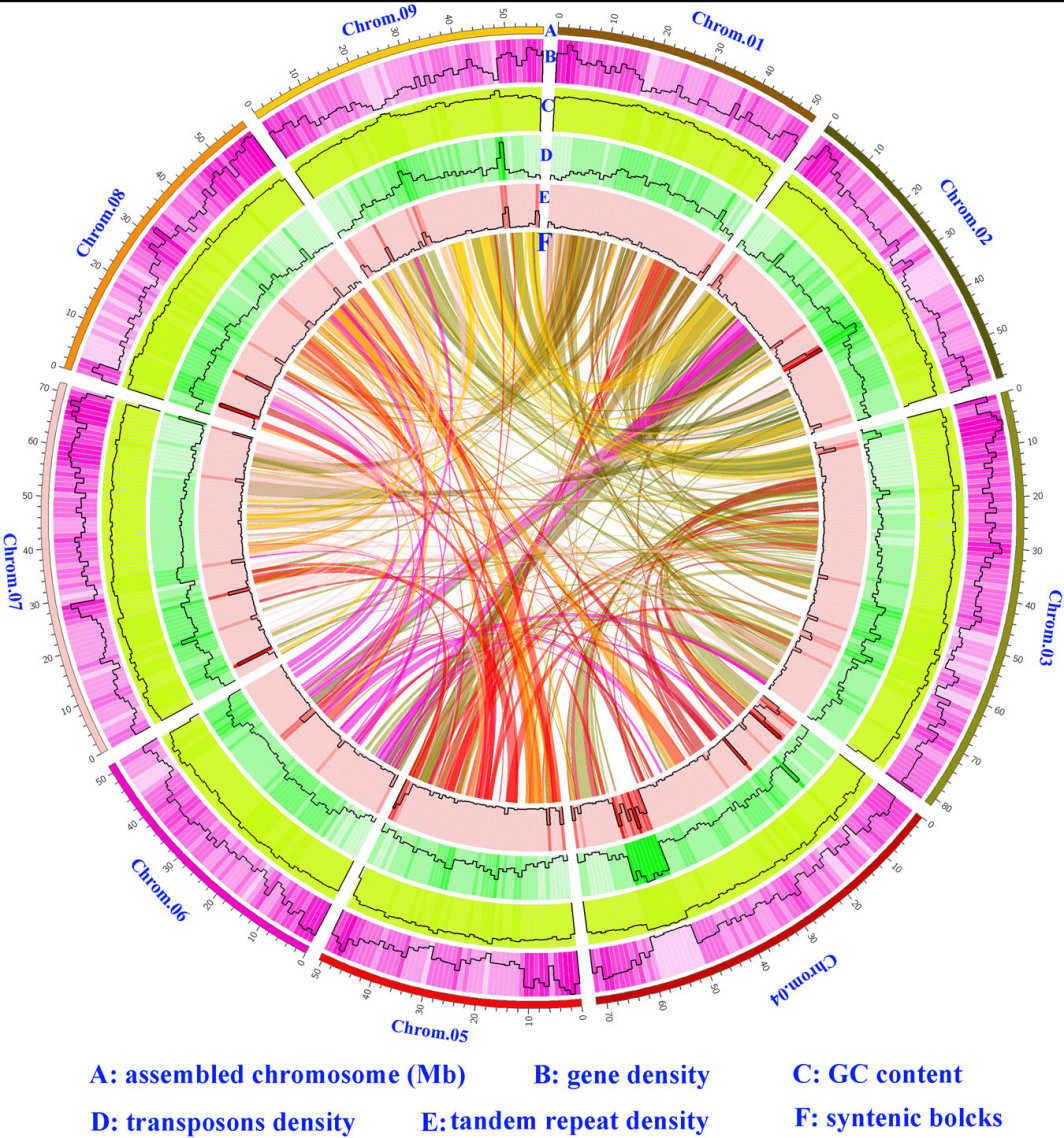
Circos diagram of different elements on the chromosomes of cauliflower. Chrom. 01, 02, 03, 04, 05, 06, 07, 08, and 09 indicate the nine assembled chromosomes of cauliflower

Synteny analysis was conducted in cauliflower and four other representative *Brassica* species, namely, *B. nigra*, *B. napus* Darmor*-bzh*, *B. oleracea TO1000DH*, and *B. rapa*, to further understand the genome evolution of cauliflower. The results indicated that 607 syntenic blocks, which involved 31,380 genes, were present between cauliflower and *B. oleracea TO1000DH* (Figure 3, Table S6 and S7). Among the 607 syntenic blocks, 103 were distributed on chromosome no. 03 of cauliflower, representing the most syntenic blocks on the 9 chromosomes, followed by 79 on chromosome no. 07; 73 on chromosome no. 04; 71 on chromosome no. 08; 61 on chromosome no. 09; 59 on chromosome no. 02; 58 on chromosome no. 06; 55 on chromosome no. 05; and 48 on chromosome no. 01. A total of 777 syntenic blocks were detected between cauliflower and *B. nigra*, which were composed of 34,361 genes (Figure 3, Table S8 and S9). Similarly, chromosome no. 03 of cauliflower shared the most syntenic blocks (122) with the eight chromosomes of *B. nigra*, followed by chromosome nos. 04 and 07. A total of 761 syntenic blocks existed between cauliflower and *B. rapa*. Among them, 126 were distributed on chromosome no. 03 of cauliflower, also representing the most syntenic blocks on the 9 chromosomes. These combined 761 syntenic blocks were composed of 39,449 genes (Figure 3, Table S10, S11). These results indicated that chromosome no. 03 of cauliflower shared the most syntenic blocks with the A and B genomes of *Brassica* species and the C genome of *B. oleracea*. Moreover, synteny analysis of the 9 chromosomes of cauliflower confirmed that chromosome no. 03 shared the most syntenic blocks with the 8 other chromosomes (Fig. 4, Table S12 and S13). These findings suggested that chromosome no. 03 is the most ancient one in the cauliflower genome, which was inherited from the common ancestor of *Brassica* species.

**Fig. 5:**
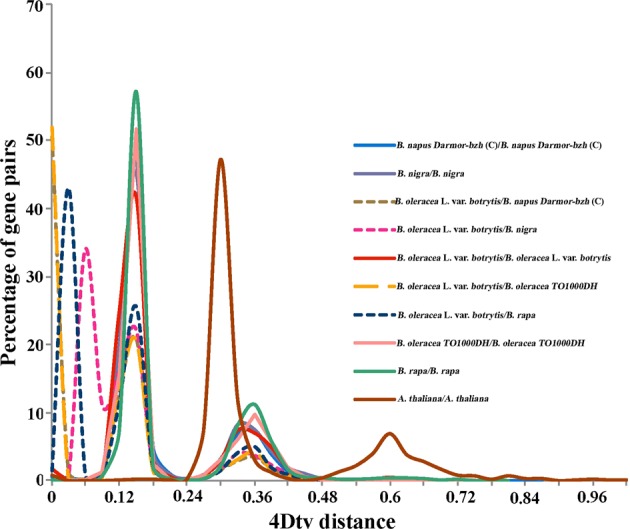
Distribution of 4DTv distances. The *x* axis indicates the 4Dtv distance. The *y* axis indicates the percentage of gene pairs. (C) shows the C subgenome of *B. napus Darmor-bzh*

According to the syntenic blocks detected in cauliflower and four other *Brassica* species, 4DTv values were estimated. The data revealed three peaks at approximately 0.15, 0.33, and 0.6 in cauliflower and four other *Brassica* species, whereas only two peaks at approximately 0.6 and 0.33 were detected in *Arabidopsis*. The peaks at approximately 0.6 and 0.33 revealed two WGD events (β and then α) shared with *Arabidopsis* after the ancient γ event in cauliflower and other *Brassica* species. The other peak at ~0.15 indicated an additional WGT event shared among *Brassica* species (Fig. 5). Moreover, the 4DTv analysis confirmed that the cauliflower genome first diverged from that of *B. nigra* (BB genome) and then diverged from that of *B. rapa* (AA genome). The genomes of cauliflower and *B. oleracea TO1000DH* and the C subgenome of *B. napus* Darmor*-bzh* arose recently and did not exhibit significant divergence compared with the A and B genomes of *Brassica* species. This finding was consistent with the phylogenetic analysis of *Brassica* and other representative *Cruciferae* species (Fig. 5).

### Specific gene families in cauliflower

Comparative genomic analysis was conducted to determine the specific genes only present in cauliflower. In three *Brassica* species with the C genome, namely, cauliflower, *B. oleracea* L. var. *capitata* and *B. oleracea TO1000DH*, 20,223 gene families were common. Compared with those in *B. oleracea* L. var. *capitata* and *B. oleracea TO1000DH*, 2,116 gene families, including 2,718 genes, were cauliflower specific (Fig. 1b, Table S14). Functional annotation results confirmed that these genes were significantly enriched in the GO terms involved in respiratory electron transport chain, ribosome, translation, ribonucleoprotein complex, structural constituent of ribosome, RNA–DNA hybrid ribonuclease activity, tryptophanyl-tRNA aminoacylation, intracellular organelle, structural molecule activity, endopeptidase inhibitor activity, and tryptophan-tRNA ligase activity (Table S15). KEGG analysis indicated that these specific genes were mainly involved in metabolic pathways such as mismatch repair, DNA replication, homologous recombination, nucleotide excision repair, oxidative phosphorylation, beta-alanine metabolism, benzoxazinoid biosynthesis and biosynthesis of siderophore-group nonribosomal peptides (Table S16). These data indicated that the cauliflower-specific gene families were mainly involved in the biosynthesis, metabolism, and modification of proteins and nucleic acids.

### Expansion and contraction of gene families in cauliflower

The expansion and contraction of the gene families in cauliflower were explored by comparing them with the gene families in *B. oleracea* L. var. *capitata* and *B. oleracea TO1000DH*. The results indicated that 6 gene families, including 228 genes, were significantly expanded in cauliflower, and these genes were mainly involved in peptidyl-prolyl *cis-trans* isomerase, armadillo repeat-containing kinesin, and senescence-associated protein (Table S17). Functional categories indicated that these expanded genes were significantly enriched in protein folding and zinc ion binding. In contrast, only 2 genes significantly contracted in cauliflower: UDP-glycosyltransferase and elongation factor 1-alpha 4. These contracted genes were significantly enriched in zeatin biosynthesis.

### Positively selected genes in cauliflower

The Ka/Ks of the single-copy genes was evaluated by using cauliflower as a foreground branch and two other related *Brassica* species (*B. oleracea* L. var. *capitata* and *B. oleracea TO1000DH*) as a background branch to identify the positively selected genes in cauliflower. A total of 1,065 candidate genes in cauliflower underwent positive selection (*P* < 0.05; Table S18). Most of them were enriched in the GO terms involved in nucleic acid binding, cellular nitrogen compound metabolism, organic cyclic compound metabolism, heterocycle metabolism, and nucleobase-containing compound metabolism (Table S19).

## Discussion

Cauliflower is a variety of *B. oleracea* and an important vegetable crop. *B. oleracea* also includes other vegetable crops, such as cabbage, broccoli, kale, brussels sprouts, collard greens, savoy, kohlrabi, and gai lan. These varieties display very high phenotypic diversity, even though they originated from the same ancestor. Cauliflower and broccoli exhibit extremely enlarged inflorescence meristems and/or flower buds, which form the major edible organ called the curd. Cabbage and brussels sprouts display extremely enlarged terminal leaf buds and lateral leaf buds, respectively. The leaves of kale and collard greens are obviously different from those of uncultivated wild cabbage, whereas kohlrabi has a dramatically enlarged stem, which is its edible organ. With the rapid development of high-throughput sequencing technologies, whole-genome sequencing can now be conducted to reveal the phenotypic diversity and evolution of nonmodel plant species. In *Brassica* species, the genomes of at least nine species or varieties, including one cultivar from *B. rapa* with the AA genome^11^, one cultivar from *B. nigra* with the BB genome^15^, three cultivars from *B. oleracea* with the CC genome^12–14^, three cultivars from *B. napus* with the AACC genome^16–18^, and one cultivar from *B. juncea* with the AABB genome^15^, have been sequenced. In the present study, the genome of another important cultivar from *B. oleracea*, cauliflower, was uncovered. A 584.60-Mb cauliflower genome assembly with 47,772 genes was reported. The repetitive sequences comprised 56.65% of the genome. The cauliflower genome was larger than the A genome of *B. rapa* (238.32 Mb)^11^, the B genome of *B. nigra* (396.9 Mb)^15^, and the A or B subgenome of *B. napus*^16–18^ and *B. juncea*^15^. The data confirmed that the cauliflower genome was also larger than that of *B. oleracea* cultivars *02–12* (539.91 Mb)^12^, *TO1000DH* (488.6 Mb)^13^, and *HDEM* (545.2 Mb)^14^ and the C subgenome of *B. napus*^16–18^. However, the number of genes in cauliflower was similar to or less than that in *Brassica* species with the C genome. These findings indicated that a high-quality cauliflower genome was generated and suggested that repetitive sequences and other noncoding sequences were more abundant in cauliflower than in other *B. oleracea* species. The present genomic data provide valuable references for understanding the formation and regulation of important agronomic traits in cauliflower and related species.

Cauliflower was domesticated from wild *B. oleracea*, which originally grew in the coastal Mediterranean. According to historical records, cauliflower was first introduced to France from Genoa, Italy, in the 16th century, but it did not commonly appear on grand tables until the time of Louis XIV. In the 18th century, cauliflower was introduced to India, and it was introduced to southern China in the middle of the 19th century. Currently, China is one of the countries that cultivates and consumes the most cauliflower. However, the origin and evolution of cauliflower in the genus *Brassica* is still ambiguous. Nevertheless, genomic data from other *Brassica* species have provided significant insight into the evolutionary relationships of plant species in the genus *Brassica* as well as in the whole *Cruciferae*. The *Brassica* and *Arabidopsis* lineages diverged approximately 20–35 Mya^60,61^. *B. rapa* and *B. oleracea* diverged from the *A. thaliana* lineage 5–9^11^ and 4.6 Mya^12^, respectively. Good estimates indicated the separation of *B. oleracea* from *B. rapa* approximately 4 Mya, and *B. nigra* separated from the A/C lineage 6–14.6 Mya^62,63^. The divergence time between *B. napus* and its progenitors was ~7500 years ago or less, based on different methods of estimation^16,17^. Consistent with previous investigations, the present cauliflower genomic data and phylogenetic analysis confirmed that the *Brassica* species with the B genome, such as *B. nigra*, are more ancient than those of *Brassica* species with the A or C genome. The *Brassica* species with the A genome and those with the B genome diverged approximately 8.5 Mya. The *Brassica* species with the A genome further diverged ~3.8 Mya, whereas those with the C genome diverged ~3.0 Mya. These results implied that the *Brassica* species with the A genome appeared earlier than those with the C genome. The C genome of the *B. oleracea* ancestral species diverged into the C subgenome of allotetraploid *B. napus* and the C genome of diploid varieties, including *B. oleracea* L. var. *capitata*, *B. oleracea TO1000DH*, and cauliflower, implying that the C subgenome in *B. napus* originated from the genome of the ancestral diploid *B. oleracea* species, while it was different from the genome of the modern diploid *B. oleracea* species. The present results also confirmed that *B. oleracea* L. var. *capitata* was generated earlier than cauliflower. The cauliflower genome was likely generated ~2.0 Mya, making cauliflower the youngest known variety of *B. oleracea* as well as in the genus *Brassica*.

Except for the specialized organ, the curd, the phenotypes of cauliflower are similar to those of closely related species in the genus *Brassica*, such as uncultivated *B. oleracea TO1000DH* and *B. oleracea* L. var. *capitata*. Especially in the seedling phase, distinguishing one species from another is difficult. Consequently, identifying the differential genes between cauliflower and closely related species is important for uncovering the phenotypic specificity of cauliflower. Here, comparative genomic analysis of cauliflower and two other *B. oleracea* species, namely, *B. oleracea TO1000DH* and *B. oleracea* L. var. *capitata*, confirmed that 2,116 gene families were cauliflower specific, while only six gene families and two gene families were significantly expanded and contracted in cauliflower, respectively. In addition, compared with the genes in *B. oleracea TO1000DH* and *B. oleracea* L. var. *capitata*, 1,065 positively selected genes were identified in cauliflower. These specific, positively selected, expanded, and contracted genes provided valuable insight into the formation of phenotypic characteristics and evolution of cauliflower. For example, several GO terms or metabolic pathways targeted by the cauliflower-specific gene families displayed significant enrichment in tryptophanyl-tRNA aminoacylation and tryptophan-tRNA ligase activity. Tryptophan is an important precursor of auxin biosynthesis^64,65^. The only two contracted gene families compared with *B. oleracea TO1000DH* and *B. oleracea* L. var. *capitata* are both involved in zeatin biosynthesis, which plays crucial roles in cytokinin biosynthesis^66,67]^. These results indicated that novel genes involved in auxin biosynthesis might have been generated during the evolution of cauliflower. However, the genes involved in cytokinin biosynthesis were contracted. Auxin and cytokinin are two important phytohormones that mainly function in regulating plant growth and development. The adjustment of the homeostasis of auxin and cytokinin in cauliflower relative to that in other *B. oleracea* species might be associated with the phenotypic characteristics of cauliflower.

In conclusion, the high-quality cauliflower genome was reported. Cauliflower underwent two WGD events and one WGT event after the ancient γ event. Chromosome no. 03 may be the most ancient one in the cauliflower genome, as it was inherited from the common ancestor of *Brassica* species. Cauliflower may be the youngest known variety in the genus *Brassica*. Moreover, specific, positively selected, expanded and contracted genes that may be closely associated with the phenotypic characteristics and evolution of cauliflower were identified. These findings provide new insight into the genomic diversity of *Brassica* species and are valuable for molecular breeding of cauliflower.

## Supplementary information

Table S1-13,S15-S16 and S19

Figure S1

Table S14

Table S17

Table S18
