# Supplementary material for: Draft genome sequence of cauliflower (Brassica oleracea L. var. botrytis) provides new insights into the C genome in Brassica species
Source: Hortic Res. 2019 Jul 1;6:82. doi: 10.1038/s41438-019-0164-0 (PMC6804732; doi:10.1038/s41438-019-0164-0)
Supplement: Supplementary file 1 — Table S1-13,S15-S16 and S19 [file 41438_2019_164_MOESM1_ESM.docx]

**Table S1** Coverage statistics of cauliflower genome by SAM tools

| Reads | Mapping rate (%) | 99.43% |
| --- | --- | --- |
| Genome | Coverage^a^ (%) | 99.15% |
|  | Coverage at least 4X (%) | 98.67% |
|  | Coverage at least 10X (%) | 98.12% |
|  | Coverage at least 20X (%) | 96.98% |
|  | Average sequencing depth^b^ | 62.58X |

^a^Coverage**:** The proportion of genomes that were covered by reads; ^b^Average sequence depth: The average depth of each base on the genome that is covered by reads.

**Table S2** Assessment of the completeness of the cauliflower genome assembly by CEGMA (Core Eukaryotic Genes Mapping Approach)

| species | Complete core genes | | (complete + partial) core genes | |
| --- | --- | --- | --- | --- |
| cauliflower | # Prots | % completeness | # Prots | % completeness |
|  | 240 | 96.77 | 243 | 97.98 |

**Table S3** Assessment of the completeness of the cauliflower genome assembly by BUSCO (Benchmarking Universal Single-Copy Orthologs)

| Species | BUSCO notation assessment results |
| --- | --- |
| cauliflower | C: 97.2% [S: 82.8%,D: 14.4%], F: 0.9%, M: 1.9%, n: 1440 |

BUSCO notation: C: Complete single-copy BUSCOs; S: Complete and single-copy BUSCOs; D: Complete duplicated BUSCOs; F: Fragmented BUSCOs; M: Missing BUSCOs; n: Total BUSCO groups searched.

**Table S4** Distribution of repeat sequences in cauliflower genome

| Types | Length (bp) | % in Genome |
| --- | --- | --- |
| TEs | 73,747,535 | 12.62 |
| LINE | 30,387,312 | 5.20 |
| SINE | 335,748 | 0.06 |
| LTR | 191,201,240 | 32.71 |
| Simple repeats | 16,918,577 | 2.89 |
| Tandem repeats | 54,625,610 | 9.34 |
| Unknown | 25,414,839 | 4.35 |

TEs: Transposable elements; LINE: Long interspersed nuclear elements; SINE: Short interspersed nuclear elements; LTR: Long terminal repeat.

**Table S5** Statistical analysis of non-coding RNAs in cauliflower genome

| Type | Number | Average length (bp) | Total length (bp) | % of genome |
| --- | --- | --- | --- | --- |
| miRNA | 1,828 | 102 | 185,819 | 0.03 |
| tRNA | 1,408 | 75 | 105,799 | 0.02 |
| rRNA | 2,420 | 315 | 762,398 | 0.13 |
| snRNA | 2,450 | 109 | 266,782 | 0.05 |

**Table S6** Syntenic blocks between cauliflower and *B. oleracea* L. var. *capitata*

| Bolb  BolC | C^a^01 | C^a^02 | C^a^03 | C^a^04 | C^a^05 | C^a^06 | C^a^07 | C^a^08 | C^a^09 |
| --- | --- | --- | --- | --- | --- | --- | --- | --- | --- |
| C^b^01 | 19 | 1 | 12 | 0 | 8 | 0 | 18 | 1 | 1 |
| C^b^02 | 0 | 16 | 7 | 1 | 0 | 9 | 8 | 0 | 14 |
| C^b^03 | 7 | 10 | 33 | 13 | 5 | 4 | 9 | 0 | 14 |
| C^b^04 | 1 | 0 | 13 | 46 | 2 | 3 | 1 | 6 | 0 |
| C^b^05 | 7 | 0 | 6 | 0 | 21 | 0 | 5 | 19 | 0 |
| C^b^06 | 0 | 10 | 9 | 3 | 0 | 38 | 3 | 7 | 1 |
| C^b^07 | 11 | 10 | 9 | 1 | 2 | 0 | 24 | 4 | 10 |
| C^b^08 | 2 | 0 | 2 | 9 | 17 | 4 | 2 | 34 | 1 |
| C^b^09 | 1 | 12 | 12 | 0 | 0 | 0 | 9 | 0 | 20 |
| Total | 48 | 59 | 103 | 73 | 55 | 58 | 79 | 71 | 61 |

Bolb: Cauliflower; BolC: *Brassica oleracea* L. var. *capitata*; C^a^01-09: The 9 chromosomes of cauliflower; C^b^01-09: The 9 chromosomes of *Brassica oleracea* L. var. *capitata.*

**Table S7** Genes involved in the syntenic blocks between cauliflower and *B. oleracea* L. var. *capitata*

| Bolb  BolC | C^a^01 | C^a^02 | C^a^03 | C^a^04 | C^a^05 | C^a^06 | C^a^07 | C^a^08 | C^a^09 |
| --- | --- | --- | --- | --- | --- | --- | --- | --- | --- |
| C^b^01 | 2662 | 10 | 215 | 0 | 130 | 0 | 305 | 11 | 6 |
| C^b^02 | 0 | 2142 | 130 | 7 | 0 | 79 | 184 | 0 | 182 |
| C^b^03 | 94 | 170 | 4017 | 176 | 70 | 38 | 103 | 0 | 250 |
| C^b^04 | 11 | 0 | 177 | 3034 | 67 | 31 | 7 | 88 | 0 |
| C^b^05 | 110 | 0 | 84 | 0 | 2360 | 0 | 47 | 366 | 0 |
| C^b^06 | 0 | 129 | 183 | 25 | 0 | 2463 | 85 | 92 | 17 |
| C^b^07 | 241 | 138 | 160 | 64 | 35 | 0 | 2949 | 40 | 136 |
| C^b^08 | 16 | 0 | 36 | 121 | 269 | 92 | 22 | 3151 | 11 |
| C^b^09 | 80 | 273 | 240 | 0 | 0 | 0 | 142 | 0 | 2807 |
| Total | 3214 | 2862 | 5242 | 3427 | 2931 | 2703 | 3844 | 3748 | 3409 |

Bolb: Cauliflower; BolC: *Brassica oleracea* L. var. *capitata*; C^a^01-09: The 9 chromosomes of cauliflower; C^b^01-09: The 9 chromosomes of *Brassica oleracea* L. var. *capitata.*

**Table S8** Syntenic blocks between cauliflower and *B. nigra*

| Bolb  Bni | C^a^01 | C^a^02 | C^a^03 | C^a^04 | C^a^05 | C^a^06 | C^a^07 | C^a^08 | C^a^09 |
| --- | --- | --- | --- | --- | --- | --- | --- | --- | --- |
| B01 | 17 | 5 | 22 | 3 | 21 | 6 | 8 | 5 | 2 |
| B02 | 11 | 18 | 16 | 5 | 1 | 11 | 14 | 7 | 15 |
| B03 | 1 | 12 | 22 | 26 | 3 | 5 | 5 | 8 | 19 |
| B04 | 0 | 4 | 14 | 33 | 7 | 15 | 4 | 22 | 3 |
| B05 | 14 | 1 | 21 | 23 | 10 | 2 | 4 | 9 | 2 |
| B06 | 2 | 6 | 5 | 5 | 11 | 9 | 16 | 11 | 13 |
| B07 | 8 | 12 | 8 | 3 | 24 | 27 | 27 | 17 | 0 |
| B08 | 7 | 17 | 14 | 4 | 5 | 1 | 16 | 12 | 21 |
| Total | 60 | 75 | 122 | 102 | 82 | 76 | 94 | 91 | 75 |

Bolb: Cauliflower; Bni: *Brassica nigra*; C^a^01-09: The 9 chromosomes of cauliflower; B01-08: The 8 chromosomes of *B. nigra.*

**Table S9** Genes involved in the syntenic blocks between cauliflower and *B. nigra*

| Bolb  Bni | C^a^01 | C^a^02 | C^a^03 | C^a^04 | C^a^05 | C^a^06 | C^a^07 | C^a^08 | C^a^09 |
| --- | --- | --- | --- | --- | --- | --- | --- | --- | --- |
| B01 | 988 | 75 | 877 | 59 | 444 | 117 | 232 | 122 | 506 |
| B02 | 1482 | 1167 | 391 | 104 | 69 | 159 | 648 | 168 | 314 |
| B03 | 12 | 319 | 2028 | 498 | 36 | 158 | 70 | 621 | 524 |
| B04 | 0 | 171 | 530 | 1478 | 481 | 874 | 131 | 730 | 119 |
| B05 | 367 | 13 | 534 | 1584 | 1165 | 22 | 79 | 295 | 114 |
| B06 | 15 | 578 | 240 | 121 | 620 | 186 | 464 | 280 | 539 |
| B07 | 201 | 303 | 619 | 82 | 885 | 1187 | 805 | 1175 | 0 |
| B08 | 288 | 592 | 579 | 100 | 130 | 11 | 1627 | 315 | 1544 |
| Total | 3353 | 3218 | 5798 | 4026 | 3830 | 2714 | 4056 | 3706 | 3660 |

Bolb: Cauliflower; Bni: *Brassica nigra*; C^a^01-09: The 9 chromosomes of cauliflower; B01-08: The 8 chromosomes of *B. nigra.*

**Table S10** Syntenic blocks between cauliflower and *B. rapa*

| Bolb  Bra | C^a^01 | C^a^02 | C^a^03 | C^a^04 | C^a^05 | C^a^06 | C^a^07 | C^a^08 | C^a^09 |
| --- | --- | --- | --- | --- | --- | --- | --- | --- | --- |
| A01 | 17 | 2 | 16 | 0 | 13 | 1 | 15 | 8 | 2 |
| A02 | 0 | 16 | 18 | 3 | 1 | 15 | 8 | 1 | 24 |
| A03 | 24 | 12 | 14 | 14 | 8 | 1 | 7 | 5 | 12 |
| A04 | 0 | 0 | 9 | 26 | 0 | 4 | 0 | 9 | 1 |
| A05 | 13 | 0 | 16 | 24 | 9 | 8 | 5 | 2 | 0 |
| A06 | 3 | 11 | 13 | 2 | 4 | 8 | 7 | 13 | 11 |
| A07 | 1 | 14 | 6 | 10 | 5 | 19 | 13 | 10 | 2 |
| A08 | 10 | 1 | 9 | 3 | 16 | 10 | 13 | 20 | 0 |
| A09 | 1 | 6 | 13 | 9 | 14 | 8 | 24 | 13 | 12 |
| A10 | 2 | 9 | 12 | 0 | 1 | 1 | 5 | 6 | 8 |
| Total | 71 | 71 | 126 | 91 | 71 | 75 | 97 | 87 | 72 |

Bolb: Cauliflower; Bra: *Brassica rapa*; C^a^01-09: The 9 chromosomes of cauliflower; A01-10: The 10 chromosomes of *B. rapa.*

**Table S11** Genes involved in the syntenic blocks between cauliflower and *B. rapa*

| Bolb  Bra | C^a^01 | C^a^02 | C^a^03 | C^a^04 | C^a^05 | C^a^06 | C^a^07 | C^a^08 | C^a^09 |
| --- | --- | --- | --- | --- | --- | --- | --- | --- | --- |
| A01 | 2756 | 36 | 337 | 0 | 253 | 80 | 386 | 124 | 15 |
| A02 | 0 | 2240 | 374 | 123 | 31 | 240 | 509 | 16 | 398 |
| A03 | 436 | 226 | 3102 | 329 | 133 | 17 | 1306 | 60 | 272 |
| A04 | 0 | 0 | 252 | 2098 | 0 | 65 | 0 | 205 | 49 |
| A05 | 255 | 0 | 300 | 1310 | 1348 | 294 | 148 | 73 | 0 |
| A06 | 20 | 321 | 587 | 35 | 810 | 433 | 1047 | 417 | 218 |
| A07 | 13 | 275 | 79 | 270 | 83 | 1815 | 904 | 222 | 29 |
| A08 | 234 | 6 | 858 | 92 | 315 | 127 | 159 | 1409 | 0 |
| A09 | 6 | 114 | 363 | 204 | 691 | 254 | 489 | 1885 | 1496 |
| A10 | 117 | 293 | 345 | 0 | 400 | 38 | 81 | 105 | 1594 |
| Total | 3837 | 3511 | 6597 | 4461 | 4064 | 3363 | 5029 | 4516 | 4071 |

Bolb: Cauliflower; Bra: *Brassica rapa*; C^a^01-09: The 9 chromosomes of cauliflower; A01-10: The 10 chromosomes of *B. rapa.*

**Table S12** Syntenic blocks in different chromosomes of cauliflower

| Bolb  Bolb | C01 | C02 | C03 | C04 | C05 | C06 | C07 | C08 | C09 |
| --- | --- | --- | --- | --- | --- | --- | --- | --- | --- |
| C01 | 0 | 3 | 7 | 1 | 6 | 0 | 5 | 4 | 1 |
| C02 | 1 | 0 | 7 | 1 | 0 | 5 | 8 | 2 | 14 |
| C03 | 14 | 5 | 0 | 14 | 8 | 2 | 14 | 2 | 9 |
| C04 | 1 | 2 | 13 | 0 | 1 | 6 | 5 | 7 | 2 |
| C05 | 4 | 2 | 7 | 0 | 0 | 6 | 5 | 10 | 2 |
| C06 | 0 | 9 | 8 | 3 | 4 | 0 | 1 | 3 | 0 |
| C07 | 8 | 4 | 13 | 2 | 5 | 1 | 0 | 6 | 9 |
| C08 | 7 | 0 | 5 | 7 | 9 | 9 | 7 | 0 | 2 |
| C09 | 2 | 8 | 15 | 0 | 2 | 2 | 11 | 0 | 0 |
| Total | 37 | 33 | 75 | 28 | 35 | 31 | 56 | 34 | 39 |

Bolb: Cauliflower; C01-09: The 9 chromosomes of cauliflower.

**Table S13** Genes involved in the syntenic blocks in different chromosomes of cauliflower

| Bolb  Bolb | C01 | C02 | C03 | C04 | C05 | C06 | C07 | C08 | C09 |
| --- | --- | --- | --- | --- | --- | --- | --- | --- | --- |
| C01 | 0 | 130 | 170 | 250 | 451 | 0 | 83 | 90 | 30 |
| C02 | 15 | 0 | 212 | 10 | 0 | 147 | 176 | 50 | 491 |
| C03 | 347 | 70 | 0 | 326 | 192 | 55 | 307 | 37 | 303 |
| C04 | 8 | 19 | 349 | 0 | 49 | 104 | 43 | 129 | 39 |
| C05 | 171 | 25 | 252 | 0 | 0 | 103 | 285 | 303 | 16 |
| C06 | 0 | 533 | 209 | 29 | 39 | 0 | 33 | 115 | 0 |
| C07 | 453 | 167 | 215 | 70 | 104 | 10 | 0 | 89 | 245 |
| C08 | 96 | 0 | 58 | 239 | 574 | 207 | 325 | 0 | 32 |
| C09 | 16 | 285 | 722 | 0 | 192 | 26 | 187 | 0 | 0 |
| Total | 1106 | 1229 | 2187 | 924 | 1601 | 652 | 1439 | 813 | 1156 |

Bolb: Cauliflower; C01-09: The 9 chromosomes of cauliflower.

**Table S15** Significantly enriched GO terms involved in the cauliflower-specific genes

| GO_ID | GO_Terms | GO_Class | Adjusted *P*-value |
| --- | --- | --- | --- |
| GO:0022904 | respiratory electron transport chain | BP | 3.67E-06 |
| GO:0006412 | translation | BP | 3.67E-06 |
| GO:0030529 | ribonucleoprotein complex | CC | 1.51E-05 |
| GO:0003735 | structural constituent of ribosome | MF | 2.88E-05 |
| GO:0005840 | ribosome | CC | 2.88E-05 |
| GO:0005198 | structural molecule activity | MF | 0.000825 |
| GO:0004523 | RNA-DNA hybrid ribonuclease activity | MF | 0.006785 |
| GO:0043229 | intracellular organelle | CC | 0.037492 |
| GO:0004866 | endopeptidase inhibitor activity | MF | 0.038281 |
| GO:0004830 | tryptophan-tRNA ligase activity | MF | 0.038281 |
| GO:0006436 | tryptophanyl-tRNA aminoacylation | BP | 0.038281 |
| GO:0004521 | endoribonuclease activity | MF | 0.049684 |

BP: Biological process; MF: Molecular function; CC: Cellular component.

**Table S16** Significantly enriched KEGG pathway involved in cauliflower-specific genes

| KEGG pathway | Adjusted *P*-value |
| --- | --- |
| Mismatch repair | 3.60E-44 |
| DNA replication | 3.15E-42 |
| Homologous recombination | 2.92E-39 |
| Fanconi anemia pathway | 3.55E-39 |
| Nucleotide excision repair | 5.92E-38 |
| Oxidative phosphorylation | 2.02E-05 |
| Benzoxazinoid biosynthesis | 0.004888 |
| beta-Alanine metabolism | 0.017971 |
| Biosynthesis of siderophore group nonribosomal peptides | 0.027475 |

**Table S19** Significantly enriched GO terms involved in the cauliflower positive selected genes

| GO_ID | GO_Term | GO_Class | Adjusted *P*-value |
| --- | --- | --- | --- |
| GO:0003676 | nucleic acid binding | MF | 0.004088 |
| GO:0034641 | cellular nitrogen compound metabolic process | BP | 0.032653 |
| GO:1901360 | organic cyclic compound metabolic process | BP | 0.032653 |
| GO:0046483 | heterocycle metabolic process | BP | 0.032653 |
| GO:0006139 | nucleobase-containing compound metabolic process | BP | 0.032653 |
| GO:0090304 | nucleic acid metabolic process | BP | 0.03423 |
| GO:0006725 | cellular aromatic compound metabolic process | BP | 0.03423 |

BP: Biological process; MF: Molecular function; CC: Cellular component.
