# Supplementary material for: Draft genome sequence of cauliflower (Brassica oleracea L. var. botrytis) provides new insights into the C genome in Brassica species
Source: Hortic Res. 2019 Jul 1;6:82. doi: 10.1038/s41438-019-0164-0 (PMC6804732; doi:10.1038/s41438-019-0164-0)
Supplement: Supplementary file 2 — Figure S1 [file 41438_2019_164_MOESM2_ESM.docx]

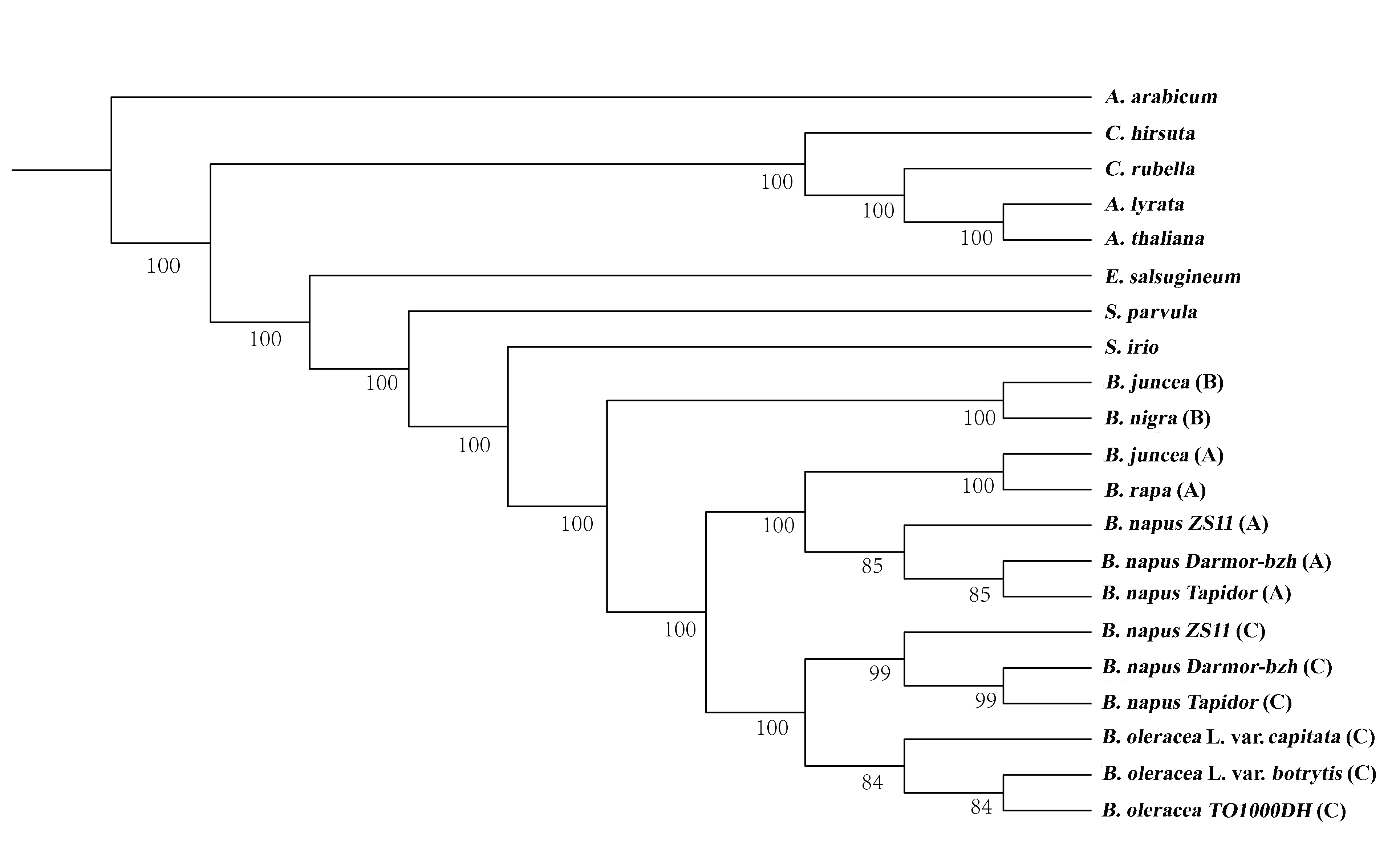


**Figure S1** Unrooted phylogenetic tree of cauliflower and other 20 representative plant genomes. The branch length indicated the evolutional ratio. The numbers on each branch indicated the bootstrap value. (A), (B) and (C) indicated the A, B and C genomes in diploid *Brassica* species: *B. rapa*, *B. oleracea* and *B. nigra*, and the subgenomes in *B. juncea* and *B. napus*.
